# ASO Author Reflections: New Persistent Benzodiazepine Use following Cancer Surgery

**DOI:** 10.1245/s10434-024-16861-x

**Published:** 2025-01-10

**Authors:** Zayed Rashid, Timothy M. Pawlik

**Affiliations:** Department of Surgery, Wexner Medical Centre, The Urban Meyer III and Shelley Meyer Chair for Cancer Research, The Ohio State University, Columbus, OH USA

## Past

Benzodiazepines rank among the most commonly prescribed psychotropic medications in high income countries and are known for their sedative, hypnotic, and anxiolytic properties.^1^ These characteristics make benzodiazepine useful among patients undergoing surgery, particularly individuals with cancer.^2^ However, the stressor characteristics of a cancer diagnosis and treatment can place patients at risk of aversive coping mechanisms.^3^ Of note, patients with newly prescribed perioperative benzodiazepines can continue to use these drugs for durations longer than initially intended.^4^ This condition is often referred to as new persistent benzodiazepine use (NPBU).^4^ Patients with cancer may be at particular risk of NPBU as they often require higher doses to manage conditions such as therapy related nausea or vomiting.^2^ As such, the current study sought to assess NPBU among patients undergoing a surgical procedure for cancer, and to identify risk factors associated with NPBU.^2^

## Present

Among 34,637 patients who underwent resection of primary cancer of breast, lung, esophagus, stomach, pancreas, liver, gall bladder, colon, rectum, or prostate, almost 8% developed NPBU. Patients with mental health illnesses, such as depression (12.7% versus 7.2%) or anxiety (15.5% versus 7.7%) were more likely to develop NPBU. Similarly, patients who underwent neoadjuvant therapy (chemotherapy: 88.1% versus 61.1%; radiotherapy: 33.8% versus 24.0%) and adjuvant therapy (chemotherapy: 79.4% versus 44.4%; radiotherapy: 17.2% versus 10.5%) more often developed NPBU. Of note, the development of concurrent new persistent use of opioids was associated with 84% higher odds of concomitant NPBU. Interestingly, patients with new perioperative benzodiazepine exposure versus non-exposure had two-fold higher odds of NPBU [odds ratio (OR) 2.00, 95% confidence interval (CI) 1.68–2.38]. This association was consistent across high-versus-low doses of perioperative benzodiazepine (ref. dose of < 10.5 LME; dose of 10.5–19.9 LME: OR 1.71, 95% CI 1.40–2.10; dose of 20.0–32.0 LME: OR 1.88, 95% CI 1.55–2.29; dose of > 32.0 LME: OR 2.42, 95% CI 2.01–2.92).

## Future

Surgical patients, particularly individuals with malignancy, can often have multiple co-morbidities and complex care needs.^3^ There can be a high prevalence of behavioral disorders (e.g., eating or sleeping disorders), as well as mental health problems (e.g., depression or anxiety) among these individuals.^3^ These morbidities in turn can lead to higher utilization of benzodiazepines following surgery and development of surgical complications, such as NPBU.^2^ As such, multidisciplinary management of these patients with counseling services can mitigate the risk of such complications.^3^ Similarly, a vast majority of patients can have leftover pills from prescriptions related to the index surgical admission.^5^ This reservoir of unneeded medications can act as a major contributor to narcotic diversion among patients.^5^ NPBU can be addressed through implementation of programs to encourage convenient and safe disposal of leftover medications.^2^ Furthermore, alternatives to benzodiazepine should be employed whenever possible. When benzodiazepines are unavoidable, these drugs should be used with lower doses and shorter durations.^2^

## References

[1] Peng L, Meeks TW, Blazes CK. Complex persistent benzodiazepine dependence—when benzodiazepine deprescribing goes awry. *JAMA Psychiatry*. 2022;79(7):639–40. 10.1001/jamapsychiatry.2022.1150. 35583897 10.1001/jamapsychiatry.2022.1150

[2] Rashid Z, Woldesenbet S, Khalil M, et al. Perioperative benzodiazepine exposure impacts risk of new persistent benzodiazepine use among patients with cancer. *Ann Surg Oncol*. 2024. 10.1245/s10434-024-16788-3. 39733079 10.1245/s10434-024-16788-3

[3] Katayama ES, Woldesenbet S, Munir MM, et al. effect of behavioral health disorders on surgical outcomes in cancer patients. *J Am Coll Surg*. 2024;238(4):625–33. 10.1097/XCS.0000000000000954. 38420963 10.1097/XCS.0000000000000954

[4] Sakamoto MR, Eguchi M, Azelby CM, et al. New persistent opioid and benzodiazepine use after curative-intent treatment in patients with breast cancer. *J Natl Compr Canc Netw*. 2021;19(1):29–38. 10.6004/jnccn.2020.7612. 33406490 10.6004/jnccn.2020.7612

[5] Khalil M, Woldesenbet S, Munir MM, et al. Surgical opioid prescription and the risk of opioid initiation among opioid-naive households. *Am J Surg*. 2025;241:116029. 10.1016/j.amjsurg.2024.116029. 39500227 10.1016/j.amjsurg.2024.116029

